# Analysis of the Pro- and Anti-Inflammatory Cytokines Secreted by Adult Stem Cells during Differentiation

**DOI:** 10.1155/2015/412467

**Published:** 2015-08-02

**Authors:** Amy L. Strong, Jeffrey M. Gimble, Bruce A. Bunnell

**Affiliations:** ^1^Center for Stem Cell Research and Regenerative Medicine, Tulane University School of Medicine, 1430 Tulane Avenue, New Orleans, LA 70112, USA; ^2^Departments of Medicine, Tulane University School of Medicine, New Orleans, LA 70112, USA; ^3^Departments of Surgery, Tulane University School of Medicine, New Orleans, LA 70112, USA; ^4^Departments of Pharmacology, Tulane University School of Medicine, New Orleans, LA 70112, USA

## Abstract

Adipose-derived stromal/stem cells (ASCs) are adult stem cells that have the potential to differentiate into mesenchymal lineage cells. The abundance of ASCs in adipose tissue and easy accessibility with relatively little donor site morbidity make them attractive candidate cells for tissue engineering and regenerative medicine. However, the underlying inflammatory process that occurs during ASC differentiation into adipocytes and osteoblast has not been extensively investigated. ASCs cultured in osteogenic and adipogenic differentiation medium were characterized by oil red o staining and alizarin red staining, respectively. ASCs undergoing osteogenic and adipogenic differentiation were isolated on days 7, 14, and 21 and assessed by qRT-PCR for the expression of pro- and anti-inflammatory cytokines. ASCs undergoing osteogenic differentiation expressed a distinct panel of cytokines that differed from the cytokine profile of ASCs undergoing adipogenic differentiation at each of the time points analyzed. Mapping the cytokine expression profile during ASC differentiation will provide insight into the role of inflammation in this process and identify potential targets that may aid in enhancing osteogenic or adipogenic differentiation for the purposes of tissue engineering and regenerative medicine.

## 1. Introduction

Adipose-derived stromal/stem cells (ASCs) are adult stem cells with multipotential differentiation capacity. The ability for ASCs to differentiate along osteogenic and adipogenic lineage cells makes them ideal candidates for regenerative medicine [1, 2]. Furthermore, the abundance and easy accessibility in harvesting large volumes of adipose tissues allow for large-scale expansion of ASCs for therapeutic purposes [3, 4].

The osteogenic and adipogenic differentiation of ASCs has been shown to require the activation of key transcriptional factors that govern cell fate. RUNX2 has previously been shown to be a master regulator of osteoblast differentiation, as RUNX2 activates and regulates many osteogenic signaling pathways, including but not limited to transforming growth factor beta (TGF-*β*), bone morphogenetic protein (BMP), Wingless type Wnt, and Hedgehog [5, 6]. ASCs cultured in osteogenic differentiation medium have also been shown to upregulate a key osteogenic factor dickkopf  Wnt signaling pathway inhibitor 1 (DKK-1), as early as one day. Additional osteogenic transcriptional factors (connective tissue growth factor (CTGF), platelet-derived growth factor receptor beta (PDGFR-*β*), TGF-*β*, insulin-like growth factor binding protein 3 (IGFBP3), and tenascin C (TNC)) were induced after 7 days in osteogenic differentiation medium [7]. In contrast, peroxisome proliferator-activated receptor gamma (PPAR*γ*) is principally regarded as the master regulator of adipogenesis, since no factor can rescue adipocyte formation when PPAR*γ* is knocked out [8]. Induction of CCAAT-enhancer-binding proteins (C/EBP*β*, C/EBP*δ*) and peroxisome proliferator-activated receptor delta (PPAR*δ*) expression occurs during early adipogenic differentiation, while fatty acid binding protein 4 (FABP4), C/EBP*α*, lipoprotein lipase (LPL), leptin, and glucose transporter 4 (GLUT4) expression is upregulated during late adipogenic differentiation [9, 10].

While many studies have explored the mechanism(s) governing ASC differentiation, few studies have investigated the associated expression of inflammatory gene expression that occurs during the differentiation of these cells. The expression profile of mRNA encoding these inflammatory cytokines may provide information regarding the mechanism governing ASC differentiation. Herein, ASCs were induced to differentiate into osteogenic and adipogenic lineage cells and assessed by qRT-PCR for the expression of pro- and anti-inflammatory cytokines. These studies demonstrated a systemic and robust upregulation of pro- and anti-inflammatory cytokines that was time-dependent. These studies demonstrated the plasticity of ASCs and identified inflammatory cytokines secreted at different stages of differentiation that may govern the ultimate cell fate of ASCs.

## 2. Materials and Methods

### 2.1. Materials

Anti-CD45-PeCy7, anti-CD11b-PeCy5, anti-CD166-phycoerythrin (PE), anti-CD105-PE, anti-CD90-PeCy5, anti-CD34-PE, isotype control fluorescein isothiocyanate (FITC) human IgG1, and isotype-control PE human IgG2a were purchased from Beckman Coulter (Indianapolis, IN). Anti-CD44-allophycocyanin (APC) was purchased from BD Biosciences (San Jose, CA). Type 1 collagenase, bovine serum albumin (BSA, fraction V), calcium chloride, cetylpyridinum chloride (CPC) dexamethasone, isobuytlmethylxanthine, indomethacin, ascorbate 2-phosphate, *β*-glycerol phosphate, alizarin red s, and oil red o were purchased from Sigma (St. Louis, MO).

### 2.2. Human Subjects

Primary human ASCs were obtained from subcutaneous abdominal adipose tissue of 3 Caucasian females (mean age 34.6 ± 8.4 and mean body mass index 22.2 ± 1.1) undergoing elective liposuction. Tissues were obtained with written informed consent under a protocol reviewed and approved by the Pennington Biomedical (Baton Rouge, LA) Institutional Review Board. Lipoaspirates were processed by incubating tissue in 0.1% type I collagenase and 1% BSA dissolved in 100 mL of phosphate buffered saline (PBS) supplemented with 2 mM calcium chloride. The mixture was placed in a 37°C shaking water bath at 75 rpm for 60 min and then centrifuged to remove oil, fat, primary adipocytes, and collagenase solution, leaving behind a pellet of cells. Cells were resuspended in medium, which consisted of Dulbecco's Modified Eagle Medium: Nutrient Mixture F-12 (DMEM/F12; Life Technologies, Grand Island, NY) and 10% fetal bovine serum (FBS; HyClone; Logan, UT), plated on 150 cm^2^ culture dishes (NUNC, Rochester, NY) and maintained in a humidified 5% CO_2_ incubator. Fresh medium was added every 2-3 days until cells achieved 80–90% confluence and were harvested with 0.25% trypsin/1 mM EDTA (Life Technologies) and cryopreserved prior to experimental use.

### 2.3. Cell Culture

Frozen vials of approximately 10^6^ ASCs were thawed, plated onto 150 cm^2^ culture dishes in 20 mL complete culture medium (CCM), which consisted of *α*-MEM (Life Technologies), 20% FBS (Atlanta Biologicals, Duluth, GA), 1% L-glutamine (Life Technologies), and 1% penicillin/streptomycin (Life Technologies), and incubated at 37°C with 5% humidified CO_2_. After 24 hours, medium was removed and adherent viable cells were washed with PBS, harvested with 0.25% trypsin/1 mM EDTA, and replated at 100 cells per cm^2^ in CCM. Medium was replaced every 3-4 days. For all experiments, cells between passages 2 and 6 were used.

### 2.4. Flow Cytometry

ASCs were harvested with 0.25% trypsin/1 mM EDTA for 3-4 minutes at 37°C. A total of 3 × 10^5^ cells were suspended in 50 *μ*L PBS and incubated with fluorescence-labeled antibodies. The samples were incubated for 30 minutes at room temperature and washed with PBS. The samples were then analyzed with Gallios Flow Cytometer (Beckman Coulter, Brea, CA) running Kaluza software (Beckman Coulter). To assay cells by forward and side scatter, FACScan was standardized with microbeads (Dynosphere uniform microspheres; Bangs Laboratories Inc.; Thermo Scientific; Waltham, MA). At least 10,000 events were analyzed and compared with isotype controls.

### 2.5. Colony Forming Unit Assay

ASCs were plated at a density of 100 cells on a 10 cm^2^ plate (NUNC) in CCM and incubated for 14 days. Plates were then rinsed with PBS and stained with 3% crystal violet (Sigma) for 30 minutes at room temperature. Plates were washed with PBS and once with tap water. Colonies that were larger than 2 mm in diameter were counted.

### 2.6. Differentiation Protocols

*Osteogenic Differentiation*. ASCs were cultured in six-well plates (NUNC) in CCM until 70% confluence. Medium was replaced with fresh osteogenic differentiation medium (ODM) consisting of 50 *μ*M ascorbate 2-phosphate, 10 mM *β*-glycerol phosphate, and 10 nM dexamethasone. After 14 days, cells were fixed in 10% formalin for 1 hour, washed with distilled water, and stained with 1% alizarin red (pH 4.1) to visualize calcium deposition in the extracellular matrix. Images were acquired at 4x magnification on an Eclipse TE200 (Nikon, Melville, NY) with Digital Camera DXM1200F (Nikon) using ACT-1 software (Nikon). For quantification, alizarin red was extracted from each well with 10% CPC and read at 584 nm (FLUOstar optima). Protein extraction with RIPA buffer (Pierce; Thermo Scientific; Waltham, MA) and protein quantification with the BCA assay (Thermo Scientific) were performed according to manufacturer's instructions. Samples were normalized to the amount of protein in each sample.

*Adipogenic Differentiation*. ASCs were cultured in six-well plates in CCM until cells achieved 70% confluence. Medium was replaced with fresh adipogenic differentiation medium (ADM) consisting of CCM supplemented with 0.5 *μ*M dexamethasone, 0.5 mM isobuytlmethylxanthine, and 50 *μ*M indomethacin. After 14 days, cells in ADM were fixed in 10% formalin for 1 hour and stained with oil red o, composed of 2 parts PBS and 3 parts 0.5% oil red o stock solution to visualize neutral lipids. Images were acquired at 10x magnification. For quantification, oil red o was extracted from each well with isopropanol and read at 544 nm (FLUOstar optima). Protein was isolated with RIPA buffer (Pierce; Thermo Scientific; Waltham, MA) and quantified with the BCA assay (Thermo Scientific) for normalization.

### 2.7. RNA Isolation, cDNA Synthesis, and Quantitative RT-PCR (qRT-PCR) Analysis

Cells were cultured in CCM, ODM, or ADM and collected after 7, 14, and 21 days. Total RNA was extracted from ASCs using the RNeasy Mini Kit (Qiagen, Valencia, CA), purified with DNase I digestion (Invitrogen) according to manufacturer's instructions, and reverse transcribed using the SuperScript VILO cDNA synthesis kit (Invitrogen) containing random primers. Quantitative real-time PCR was performed using the EXPRESS SYBR GreenER qPCR SuperMix Kit (Invitrogen) according to the manufacturer's instructions. Forward and reverse primer sequences can be found in Table 1. All qRT-PCR primers were designed using Primer3 (Boston, MA) and purchased from Integrated DNA Technologies (Coralville, IA). The expression of human *β*-actin was used to normalize mRNA content. Samples were tested in triplicate. No-template controls and no-reverse transcription controls were included in each PCR run.

## 3. Results

### 3.1. Characterization of ASCs

**Figure 1:**
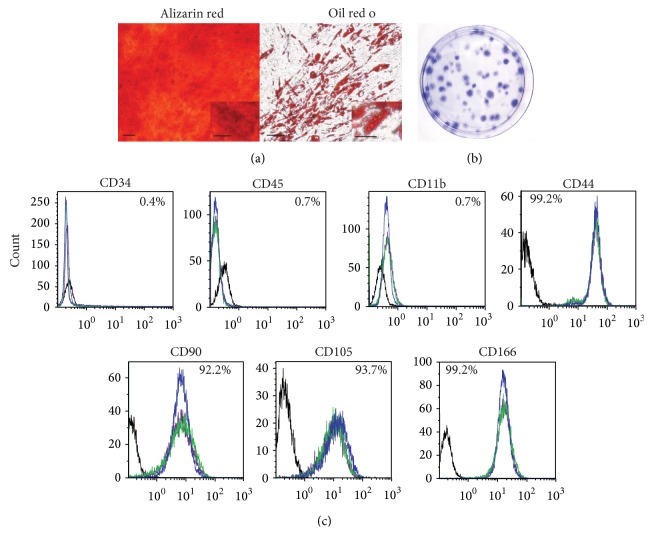
Characterization of ASCs. (a) ASCs were cultured in ODM or ADM for 21 days and stained with alizarin red for osteogenesis or oil red o for adipogenesis. Representative images are shown. Original magnification for osteogenesis is 4x and that of adipogenesis is 10x. Scale bar represents 100 *μ*m. Scale bar for insets represents 25 *μ*m. (b) Cells were seeded at low density and incubated in CCM. After 14 days, colony-forming units were stained with crystal violet. A representative image is shown. (c) ASCs were stained with antibodies against the indicated antigens and analyzed by flow cytometry. Each colored line represents a specific donor (*N* = 3 donors), and respective isotype controls are shown as black lines.

ASCs were isolated from processed lipoaspirates harvested from subcutaneous adipose tissue and characterized based on differentiation potential, self-renewal capacity, and cell surface marker profile. ASCs were able to differentiate into osteoblast and adipocytes when induced with ODM and ADM, respectively (Figure 1(a)). ASCs seeded at low density were able to generate colony-forming units (Figure 1(b)). Flow cytometric analysis demonstrated that ASCs were negative for CD34, CD45, and CD11b expression and positive for CD44, CD90, CD105, and CD166 expression (Figure 1(c)).

### 3.2. Expression of Proinflammatory and Anti-Inflammatory Cytokines during Osteogenic Differentiation of ASCs Is Time Dependent

**Figure 2:**
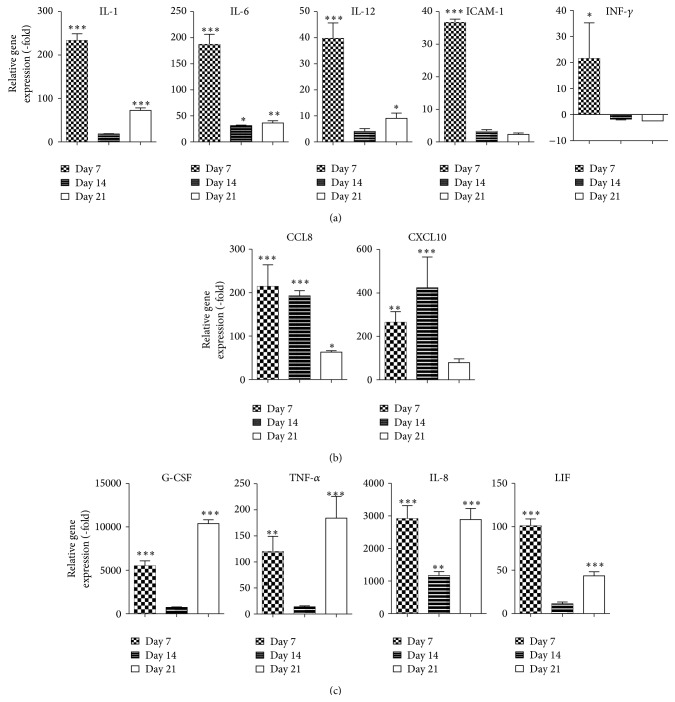
Proinflammatory cytokines are upregulated during osteogenic differentiation of ASCs. ASCs were cultured in CCM and changed to ODM. Cells were harvested on days 7, 14, or 21 and analyzed by qRT-PCR. Data is normalized to undifferentiated cells. Mean ± SEM. ^*^ *P* < 0.05; ^**^ *P* < 0.01; ^***^ *P* < 0.001 relative to undifferentiated cells.

ASCs at passage 2 were expanded in CCM until confluent and cultured in ODM. At 7, 14, and 21 days of culture, cells were harvested and the mRNA expression of pro- and anti-inflammatory cytokines was assessed in comparison to undifferentiated ASCs. ASCs induced with ODM demonstrated an increase in the expression of proinflammatory cytokines interleukin-1 (IL-1; 223.3-fold, *P* < 0.001), interleukin-6 (IL-6; 187.3-fold, *P* < 0.001), interleukin-12 (IL-12; 39.8-fold, *P* < 0.001), intercellular adhesion molecule 1 (ICAM-1; 36.7-fold, *P* < 0.001), and interferon gamma (IFN-*γ*; 21.5-fold, *P* < 0.05) during the early stages of osteogenic differentiation (day 7), and their expression diminished during the mid and late stages of differentiation (Figure 2(a)). Early and mid stages of osteogenic differentiation of ASCs demonstrated an increase in chemokine (C-C motif) ligand 8 (CCL8; 214.4-fold on day 7 and 192.5-fold on day 14, *P* < 0.001) and C-X-C motif chemokine 10 (CXCL10; 264.5-fold on day 7 and 423.9-fold on day 14, *P* < 0.001) expression, which decreased by day 21 (day 21; Figure 2(b)). In contrast, mRNA expression of several proinflammatory cytokines demonstrated a biphasic increase on day 7 and day 21, with minimal induction on day 14: granulocyte-colony stimulating factor (G-CSF) mRNA expression was increased by 5507.2-fold and 10381.3-fold, tumor necrosis factor alpha (TNF-*α*) was increased by 119.8-fold and 184.1-fold, interleukin-8 (IL-8) was increased by 2915.0-fold and 2888.3-fold, and leukemia inhibitory factor (LIF) was increased by 101.1-fold and 43.6-fold on day 7 and day 21, respectively (Figure 2(c)).

**Figure 3:**
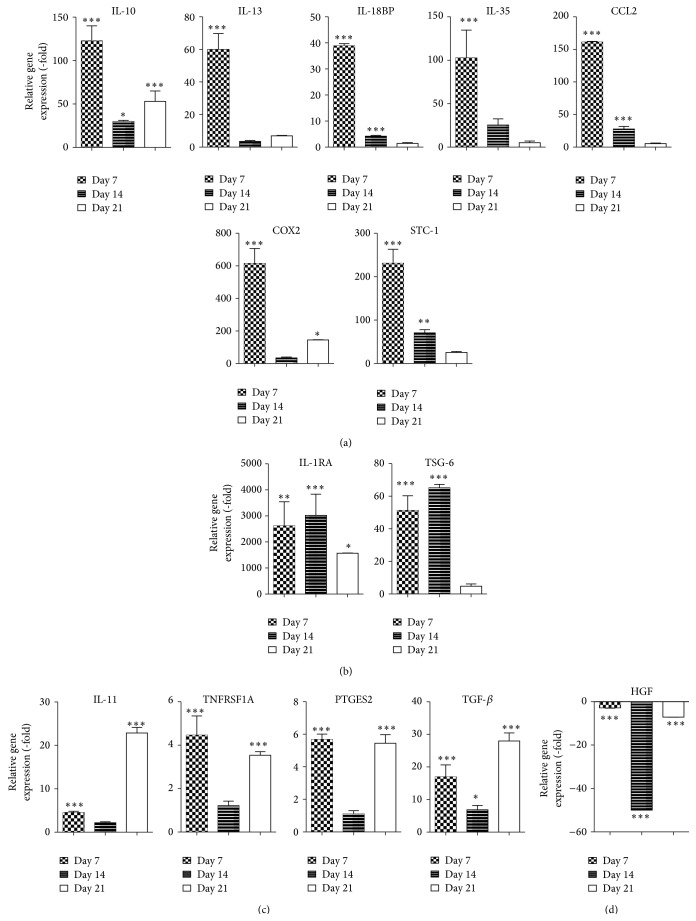
Osteogenic differentiation of ASCs increases expression of anti-inflammatory cytokines. ASCs were grown in CCM and then changed to ODM. After 7, 14, and 21 days, cells were harvested and analyzed by qRT-PCR. Data is normalized to undifferentiated cells. Mean ± SEM. ^*^ *P* < 0.05; ^**^ *P* < 0.01; ^***^ *P* < 0.001 relative to undifferentiated cells.

The expression of anti-inflammatory cytokines was also assessed during osteogenic differentiation. Of the 14 anti-inflammatory cytokines assessed, seven genes were significantly upregulated in ASCs following culture in ODM for 7 days, which diminished by days 14 and 21 after induction. Increased expression of interleukin-10 (IL-10; 122.8-fold, *P* < 0.001), interleukin-13 (IL-13; 59.8-fold, *P* < 0.001), interleukin-18 binding protein (IL-18BP; 38.9-fold, *P* < 0.001), interleukin-35 (IL-35; 102.7-fold, *P* < 0.001), chemokine (C-C motif) ligand 2 (CCL2; 161.2-fold, *P* < 0.001), cyclooxygenase 2 (COX2; 614.4-fold, *P* < 0.001), and stanniocalcin 1 (STC-1; 230.8-fold, *P* < 0.001, Figure 3(a)) was observed. In contrast, ASCs cultured in ODM for 7 and 14 days demonstrated a sustained increase in the expression of interleukin-1 receptor antagonist (IL-1RA; 2617.7-fold on day 7 and 3024.7-fold on day 14, *P* < 0.001) and tumor necrosis factor-stimulated gene 6 (TSG-6; 51.2-fold on day 7 and 65.2-fold on day 14, *P* < 0.001; Figure 3(b)). Cells cultured in ODM demonstrated a biphasic induction in mRNA expression of anti-inflammatory cytokine interleukin-11 (IL-11; 4.5-fold on day 7 and 22.9-fold on day 21, *P* < 0.001), tumor necrosis factor receptor superfamily member (TNFRSF1A; 4.5-fold on day 7 and 3.5-fold on day 21, *P* < 0.001), prostaglandin E synthase 2 (PTGES2; 5.7-fold on day 7 and 5.5-fold on day 21, *P* < 0.001), and TGF-*β* (17.0-fold on day 7 and 28.0-fold on day 21, *P* < 0.001; Figure 3(c)). In contrast, mRNA expression of hepatocyte growth factor (HGF) was reduced by −2.9-fold, −50.0-fold, and −7.1-fold on days 7, 14, and 21, respectively (*P* < 0.001; Figure 3(d)).

### 3.3. Proinflammatory and Anti-Inflammatory Cytokine Expression during Adipogenic Differentiation of ASCs Vary

**Figure 4:**
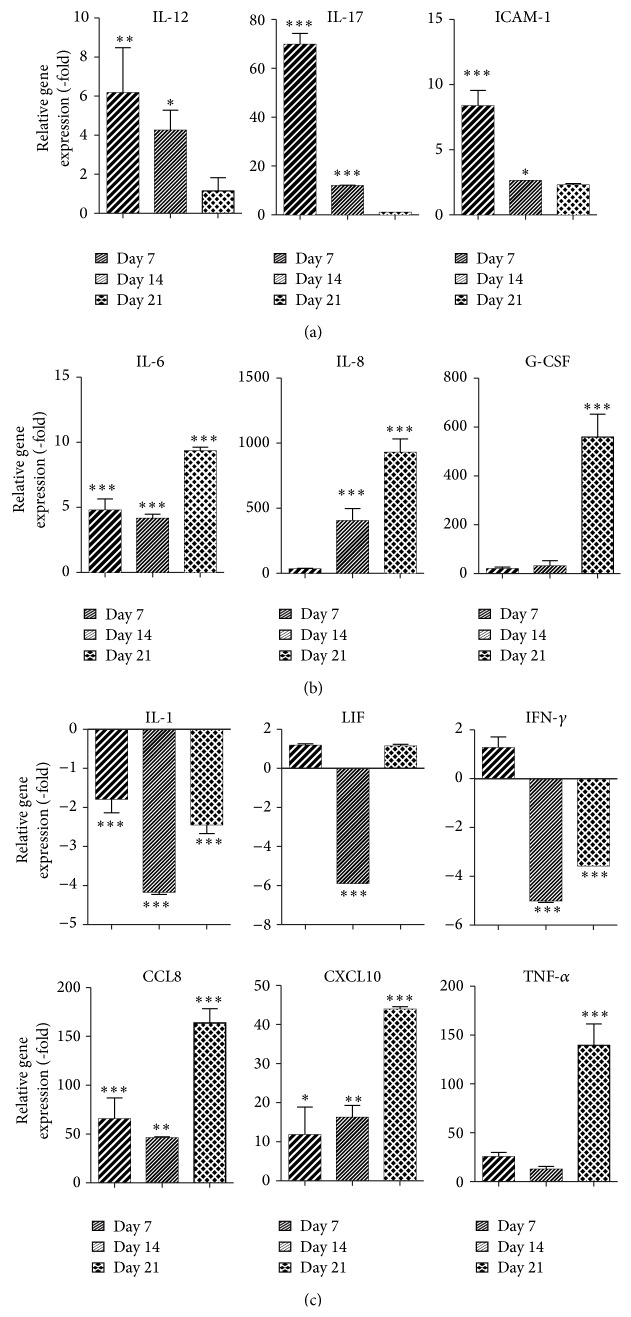
Levels of mRNA expression of proinflammatory cytokines are upregulated during adipogenic differentiation of ASCs. ASCs were grown in CCM and changed to ADM. Cells were harvested on days 7, 14, and 21 and analyzed by qRT-PCR. Data is normalized to undifferentiated cells. Mean ± SEM. ^*^ *P* < 0.05; ^**^ *P* < 0.01; ^***^ *P* < 0.001 relative to undifferentiated cells.

ASCs were expanded in CCM until confluent at which time ADM was added to the cells. Again, at 7, 14, and 21 days, cells were harvested and the mRNA expression of proinflammatory and anti-inflammatory cytokines was assessed relative to naive ASCs. ASCs induced with ADM for 7 days and 14 days demonstrated an increase in the expression of proinflammatory cytokines IL-12 (223.3-fold on day 7 and 4.3-fold on day 14, *P* < 0.05), interleukin-17 (IL-17; 69.8-fold on day 7 and 11.9-fold on day 14, *P* < 0.001), and ICAM-1 (8.4-fold on day 7 and 2.6-fold on day 14, *P* < 0.05, Figure 4(a)). In contrast, several proinflammatory cytokines demonstrated the highest levels of mRNA expression on day 21: mRNA levels for IL-6 were increased by 9.4-fold, IL-8 was increased by 929.5-fold, G-CSF was increased by 559.4-fold, CCL8 was increased by 163.8-fold, CXCL10 was increased by 43.9-fold, and TNF-*α* was increased by 139.5-fold (Figure 4(b), *P* < 0.001). In contrast, the mRNA expression of several proinflammatory cytokines was significantly reduced during adipogenic differentiation of ASCs: IL-1 (−4.2-fold on day 14 and −2.4-fold on day 21, *P* < 0.001), LIF (−5.8-fold on day 14, *P* < 0.001), and IFN-*γ* (−5.0-fold on day 14 and −3.6-fold on day 21, *P* < 0.001).

**Figure 5:**
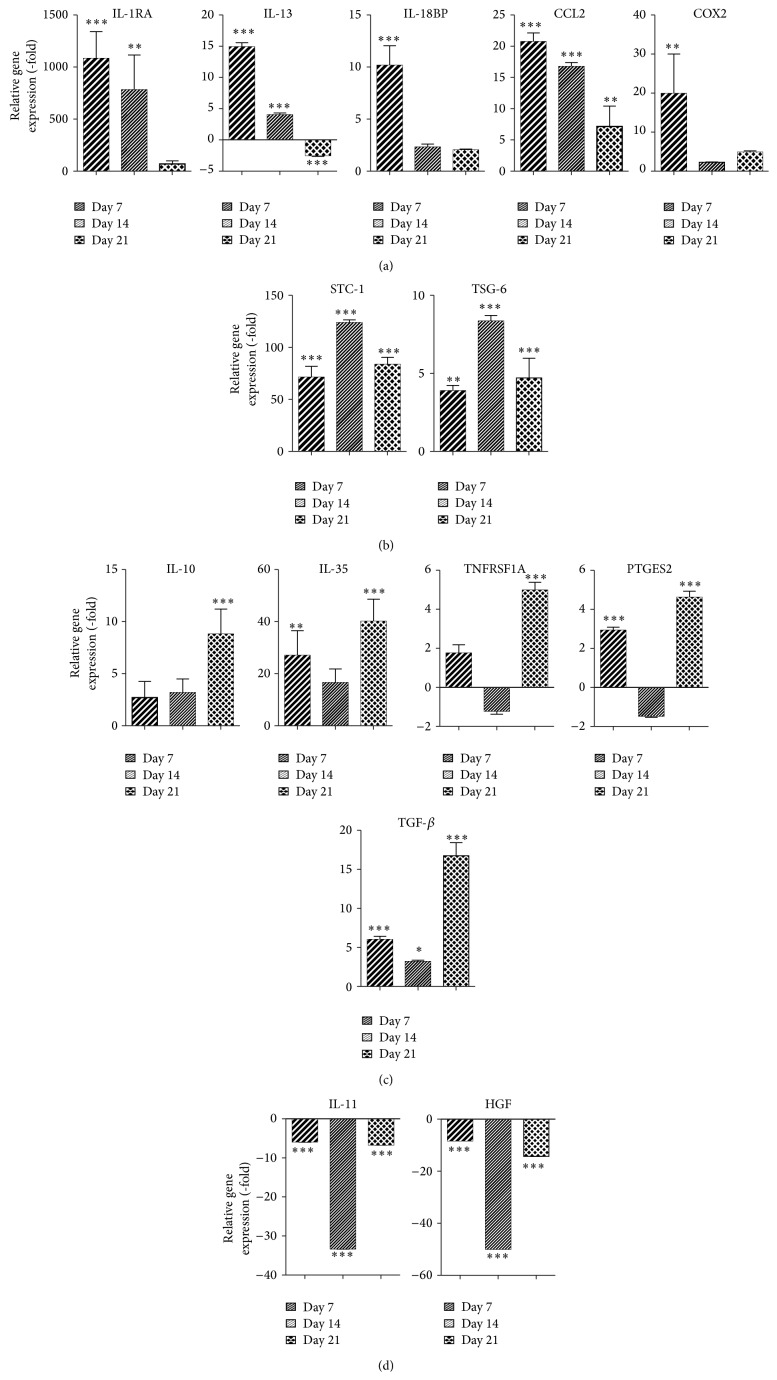
Levels of anti-inflammatory cytokines are elevated during adipogenic differentiation of ASCs. ASCs were grown in CCM and then changed to ADM. After 7, 14, and 21 days, cells were harvested and analyzed by qRT-PCR. Data is normalized to undifferentiated cells. Mean ± SEM. ^*^ *P* < 0.05; ^**^ *P* < 0.01; ^***^ *P* < 0.001 relative to undifferentiated cells.

The analysis of anti-inflammatory cytokines expressed during adipogenic differentiation demonstrated significant differences in induction level that was time dependent. ADM increased gene expression of IL-1RA (1082.6-fold, *P* < 0.001), IL-13 (14.9-fold, *P* < 0.001), IL-18BP (10.2-fold, *P* < 0.001), CCL2 (20.8-fold, *P* < 0.001), and COX2 (4.8-fold, *P* < 0.001) on day 7 (Figure 5(a)). In contrast, the mRNA expression of STC-1 and TSG6 was most significantly increased by 123.6-fold and 8.4-fold, respectively, on day 14 (*P* < 0.001, Figure 5(b)). Adipogenic differentiation of ASCs resulted in the most robust induction of IL-10 (8.8-fold, *P* < 0.001), IL-35 (40.1-fold, *P* < 0.001), TNFRSF1A (5.0-fold, *P* < 0.001), PTGES2 (4.6-fold, *P* < 0.001), and TGF-*β* (16.7-fold, *P* < 0.001, Figure 5(c)) on day 21. IL-11 and HGF mRNA expression were most significantly reduced on day 14 by −33.3-fold and −14.3-fold, respectively (*P* < 0.001, Figure 5(d)).

## 4. Discussion

The interest in ASCs for tissue engineering purposes and regenerative medicine has grown significantly due to their accessibility, abundance, and capacity to differentiate into mesenchymal lineage cells. While studies have begun to investigate the mechanism by which ASCs differentiate into adipogenic or osteogenic lineage cells, the precise role of inflammatory cytokines has not been explored extensively. The mRNA levels of many pro- and anti-inflammatory cytokines expressed by ASCs during osteogenic and adipogenic differentiations were assessed. ASCs undergoing osteogenic differentiation expressed a distinct panel of cytokines that differed from the cytokine profile of ASCs undergoing adipogenic differentiation at each of the time intervals analyzed.

**Figure 6:**
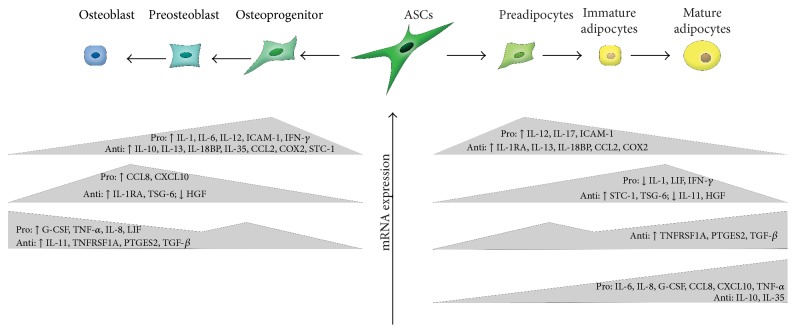
Schematic of ASCs undergoing osteogenic or adipogenic differentiation. Expression of proinflammatory and anti-inflammatory cytokines is dependent on the stage of differentiation.

A quantitative comparison of the proinflammatory cytokines and anti-inflammatory cytokines expressed during osteogenic differentiation of ASCs demonstrates three distinct groups of cytokines. These groups of cytokines are categorized based on their induction at early, mid, and late stages of osteogenic differentiation (Figure 6). While the current study investigated the inflammatory cytokines secreted during the osteogenic differentiation of these cells in a cocktail of growth factors, others have taken the approach of treating progenitor cells with a similar cocktail of growth factors and supplemented the medium with additional inflammatory cytokines, such as TNF-*α* and IL-1 [11–14]. Most of these studies were conducted in bone marrow-derived mesenchymal stem cells (BMSCs), which are derived from the mesodermal lineage and have a similar differentiation potential as ASCs. Human BMSCs treated with TNF-*α* resulted in the activation of NF-*κ*B, leading to increased mineralization and enhanced expression of osteogenic proteins, such as BMP2 and alkaline phosphatase, and transcription factors such as RUNX2 and Osterix [11, 12]. Human BMSCs treated with IL-1, likewise, enhanced differentiation into osteoblasts through the Wnt-5a/receptor tyrosine kinase-like orphan receptor 2 pathway [13]. Ferreira et al. found that IL-1 also enhanced mineralization through both nuclear factor kappa-light-chain-enhancer of activated B cells (NF-*κ*B) and mitogen-activated protein kinase (MAPK) pathways [14]. The results presented in the current study are consistent with previously published reports showing an upregulation of TNF-*α* and IL-1 during differentiation. The present study supplements the current body of literature and highlights specific inflammatory factors that should be investigated further based on their induction during osteogenic differentiation. Additional studies, however, are necessary to elucidate the precise mechanism by which these cytokines effect osteogenic differentiation.

Furthermore, it should be noted that the effects of pro- and anti-inflammatory cytokines on osteogenesis might be determined by the physical location. For instance, cytokines have been shown to contribute to a decrease in bone mineral density by inhibiting osteoblast proliferation and differentiation and enhancing the rate of osteoclast differentiation in patients with severe inflammatory disease [15, 16]. Thus, patients diagnosed with such diseases as rheumatoid arthritis and osteoarthritis have a higher incidence of osteoporosis [15, 16]. In contrast, inflammatory cytokines are strongly suspected to induce ectopic bone formation, for instance, in arteries during atherosclerosis or in postburn heterotopic ossification [17, 18]. Consistent with these observations, anti-inflammatory drugs have been shown to reduce the incidence and severity of ectopic bone formation [19].

With respect to adipogenesis, the current study demonstrated a time-dependent expression of cytokines in ASCs during adipogenic differentiation (Figure 6). While limited studies have been conducted on the cytokine profile of ASCs undergoing adipogenic differentiation, it has been previously shown that cytokines such as IL-1, IL-6, and TNF-*α* have the ability to inhibit adipogenic differentiation of BMSCs. PPAR*γ* is suppressed by IL-1 and TNF-*α*, and this suppression is mediated through NF-*κ*B [20]. In the context of obesity, increased proinflammatory cytokines are secreted by the adipose tissue due to tissue hypoxia that results from hypertrophy and hyperplasia of adipocytes [21]. Based on previous reports, this increase in proinflammatory cytokines, such as IL-1 and TNF-*α*, should result in decreased adipogenesis. However, due to the obesity-associated dysregulation of adipocytes and ASCs, these cells no longer respond to these cytokines properly [22]. Thus, the increase in IL-1 and TNF-*α* does not inhibit adipogenesis. Additional studies focusing on the impact of other pro- and anti-inflammatory cytokines on adipogenesis will shed light on ASC differentiation for the purposes of soft tissue reconstruction. Furthermore, they may provide insight into what other factors mediate adipogenesis in the context of obesity.

## 5. Conclusion

The osteogenic and adipogenic differentiation of ASCs alter the expression of an array of cytokines. The levels of induction of these pro- and anti-inflammatory cytokines are dependent on the stage (early, middle, or late) and type (osteogenic or adipogenic) of differentiation. The data presented here provides a framework for understanding the role that cytokine expression may play in tissue engineering projects using ASCs. By understanding which cytokines are upregulated during osteogenic or adipogenic differentiation, it will be possible to specifically target these molecules to enhance osteogenic or adipogenic differentiation for soft tissue defects or bone defects. Furthermore, paracrine expression of these cytokines by differentiating ASCs may change the local environment. By understanding the local environment produced by differentiation ASCs, we will also be better equipped to assess the engraftment and survival of tissue engineered constructs prepared with ASCs.

## Figures and Tables

**Table 1:** qRT-PCR primer sequences for proinflammatory and anti-inflammatory cytokines.

Gene	Forward (5′→3′)	Reverse (5′→3′)
Proinflammatory cytokines		
IL-1	CGCCAATGACTCAGAGGAAG	AGGGCGTCATTCAGGATCAA
IL-6	GTAGCCGCCCCACACAGACAGCC	GCCATCTTTGGAAGGTTC
IL-8	GAACTGAGAGTGATTGAGAGT	CTTCTCCACAACCCTCTG
IL-12	TGGAGTGCCAGGAGGACAGT	TCTTGGGTGGGTCAGGTTTG
IL-17	TGAAGGCAGGAATCACAAT	GGTGGATCGGTTGTAGTAAT
CCL8	CACAAGAATCACCAACATCC	TGGTCCAGATGCTTCATG
CXCL10	TCTGACTCTAAGTGGCATTC	ATTGTAGCAATGATCTCAACAC
G-CSF	AGCTTCCTGCTCAAGTGC	TTCTTCCATCTGCTGCCAGATGGT
ICAM-1	CACAGTCACCTATGGCAA	CTGGCTTCGTCAGAATCA
IFN-*γ*	TCAGCTCTGCATCGTTTTGG	GTTCCATTATCCGCTACATCTGAA
LIF	CCTGGACAAGCTATGTGG	GGTTGAGGATCTTCTGGTC
TNF-*α*	TCTTCTCGAACCCCGAGTGA	CCTCTGATGGCACCACCAG

Anti-inflammatory cytokines		
IL-1RA	GTTCCATTCAGAGACGATCT	GTTGTTCCTCAGATAGAAGGT
IL-10	GTGATGCCCCAAGCTGAGA	CACGGCCTTGCTCTTGTTTT
IL-11	GGACCACAACCTGGATTC	GCAGGTAGGACAGTAGGT
IL-13	ATTGCTCTCACTTGCCTT	GTCAGGTTGATGCTCCAT
IL-18BP	ACCATGAGACACAACTGG	ATGCTGGACACTGCTTAG
IL-35	CACGTCCTTCATCCTCAG	GACTCCAGTCACTCAGTTC
CCL2	AGTCACCTGCTGTTATAACTT	CACAATGGTCTTGAAGATCAC
COX2	ACAGTCCACCAACTTACAAT	CAATCATCAGGCACAGGA
HGF	TTATCCTGACGTAAACACCTTTGATATAAC	CTGGGCAGTATTCGGGTTTGA
PTGES2	CCTGGAAGAGATCATCACC	CCTTCTCGTTGAGCATGA
STC-1	AGGATGATTGCTGAGGTG	TGTTATAGTATCTGTTGGAGAAGT
TGF-*β*	CAGCAACAATTCCTGGCGATA	AAGGCGAAAGCCCTCAATTT
TNFRSF1A	CAGGAAGAACCAGTACCG	TTCTTACAGTTACTACAGGAGAC
TSG-6	CATCTCGCAACTTACAAGC	AGACGGATTCCATAATCAATAATG

## Abbreviations

ADM: Adipogenic differentiation medium
APC: Allophycocyanin
ASCs: Adipose-derived stromal/stem cells
BSA: Bovine serum albumin
BMP: Bone morphogenic protein
C/EBP: CCAAT-enhancer-binding proteins
C/EBP*α*: CCAAT-enhancer-binding proteins alpha
C/EBP*β*: CCAAT-enhancer-binding proteins beta
C/EBP*δ*: CCAAT-enhancer-binding proteins delta
CCL2: Chemokine (C-C motif) ligand 2
CCL8: Chemokine (C-C motif) ligand 8
CCM: Complete culture medium
COX2: Cyclooxygenase 2
CPC: Cetylpyridinium chloride
CTGF: Connective tissue growth factor
CXCL10: C-X-C motif chemokine 10
DKK-1: Dickkopf WNT signaling pathway inhibitor 1
DMEM/F-12: Dulbecco's Modified Eagle Medium: Nutrient Mixture F-12
FABP4: Fatty acid binding protein 4
FBS: Fetal bovine serum
FITC: Fluorescein isothiocyanate
G-CSF: Granulocyte-colony stimulating factor
GLUT4: Glucose transporter 4
HGF: Hepatocyte growth factor
IGFBP3: Insulin-like growth factor binding protein 3
ICAM-1: Intercellular adhesion molecule 1
IFN-*γ*: Interferon gamma
IL-1: Interleukin-1
IL-1RA: Interleukin-1 receptor antagonist
IL-6: Interleukin-6
IL-8: Interleukin-8
IL-10: Interleukin-10
IL-11: Interleukin-11
IL-12: Interleukin-12
IL-13: Interleukin-13
IL-17: Interleukin-17
IL-18BP: Interleukin-18 binding protein
IL-35: Interleukin-35
LIF: Leukemia inhibitory factor
LPL: Lipoprotein lipase
MAPK: Mitogen-activated protein kinase
NF-*κ*B: Nuclear factor kappa-light-chain-enhancer of activated B cells
ODM: Osteogenic differentiation medium
PBS: Phosphate buffered saline
PDGFR-*β*: Platelet-derived growth factor receptor beta
PE: Phycoerythrin
PPAR*δ*: Peroxisome proliferator-activated receptor delta
PPAR*γ*: Peroxisome proliferator-activated receptor gamma
PTGES2: Prostaglandin E synthase 2
RUNX2: Runt-related transcription factor 2
STC-1: Stanniocalcin 1
TGF-*β*: Transforming growth factor beta
TNF-*α*: Tumor necrosis factor alpha
TNFRSF1A: Tumor necrosis factor receptor superfamily member 1A
TSG-6: Tumor necrosis factor-stimulated gene 6
Wnt: Wingless type.
